# Nonconvulsive status epilepticus characteristics in glioma patients: a retrospective study

**DOI:** 10.1007/s12672-023-00632-3

**Published:** 2023-03-07

**Authors:** Azumi Kaneoka, Satoka Hashimoto Fujimoto, Kaoru Tamura, Motoki Inaji, Taketoshi Maehara

**Affiliations:** Department of Neurosurgery, Tokyo Medical and Dental University, 1-5-45 Yushima, Bunkyo-ku, Tokyo, Japan

**Keywords:** Glioma, Epilepsy, Nonconvulsive status epilepticus (NCSE), Modified Salzburg Consensus Criteria (mSCC)

## Abstract

**Purpose:**

Epilepsy is a common complication of gliomas. The diagnosis of nonconvulsive status epilepticus (NCSE) is challenging because it causes impaired consciousness and mimics glioma progression. NCSE complication rate in the general brain tumor patient population is approximately 2%. However, there are no reports focusing on NCSE in glioma patient population. This study aimed to reveal the epidemiology and features of NCSE in glioma patients to enable appropriate diagnosis.

**Methods:**

We enrolled 108 consecutive glioma patients (45 female, 63 male) who underwent their first surgery between April 2013 and May 2019 at our institution. We retrospectively investigated glioma patients diagnosed with tumor-related epilepsy (TRE) or NCSE to explore disease frequency of TRE/NCSE and patient background. NCSE treatment approaches and Karnofsky Performance Status Scale (KPS) changes following NCSE were surveyed. NCSE diagnosis was confirmed using the modified Salzburg Consensus Criteria (mSCC).

**Results:**

Sixty-one out of 108 glioma patients experienced TRE (56%), and five (4.6%) were diagnosed with NCSE (2 female, 3 male; mean age, 57 years old; WHO grade II 1, grade III 2, grade IV 2). All NCSE cases were controlled by stage 2 status epilepticus treatment as recommended in the Clinical Practice Guidelines for Epilepsy by the Japan Epilepsy Society. The KPS score significantly decreased after NCSE.

**Conclusion:**

Higher prevalence of NCSE in glioma patients was observed. The KPS score significantly decreased after NCSE. Actively taking electroencephalograms analyzed by mSCC may facilitate accurate NCSE diagnosis and improve the activities of daily living in glioma patients.

**Supplementary Information:**

The online version contains supplementary material available at 10.1007/s12672-023-00632-3.

## Introduction

Glioma is the most common primary malignant brain tumor in adults, although the incidence is low (about 5 per 100,000 persons) [1]. Survival rates for gliomas vary by glioma subtype and grade, but glioblastoma, the most malignant type with a 5-year overall relative survival rate of only 6.8%, accounts for more than half of all gliomas. [2]. Therefore, it is important to control glioma comorbidities in order to maintain activities of daily living (ADL).

Tumor-related epilepsy (TRE) is a common complication of brain tumors as forty to 80% of patients with brain tumors present with epileptic seizures at least once in all disease courses [3]. Epilepsy is one the most common brain disorders defined by recurrent epileptic seizures, affecting more than 1 in 100 people [4]. Primary brain tumors are a leading cause of epilepsy, accounting for 5% of new-onset epilepsy in adults and more than 10% of focal epilepsy [5]. A recent study also revealed that more than 70% of brain tumor patients suffer from epilepsy at the end of life [6]. Brain tumor patients with epilepsy show the higher risk of lower quality of life in addition to the morbidities and mortality [7]. For example, the most common reason reported for hospital readmission after craniotomy for malignant supratentorial tumors is new onset seizures [8].

Status epilepticus (SE), which is the most extreme form of epileptic seizures, in addition to being a neurological emergency [9]. The incidence of SE is reported to be ten to twenty per 100,000 persons per year [10]; mortality from SE is reported to be 1.9 to 40% [11].

Nonconvulsive status epilepticus (NCSE) has recently gained increased attention because of its reported high prevalence. NCSE is a subtype of status SE and generally accounts for 25 to 50% of SE [12]; although the number of studies of NCSE is still small, but the mortality rates of approximately 30% have been reported in the elderly people [13]. The feature of NCSE is lacking of prominent motor symptoms and primarily presents with impaired consciousness. For instance, language disturbance are common manifestations of non-convulsive seizures and can be easily misdiagnose as other neurological disease [14]. Accordingly, the diagnosis of NCSE is challenging, especially in patients with glioma, who frequently show impaired consciousness due to tumor progression, other comorbidities such as hemorrhage in the tumor, and treatment effects, including radiotherapy and chemotherapy [15]. To the best of our knowledge, there are no prior reports about NCSE that focused on the glioma patient population; therefore, it is difficult to diagnose and manage NCSE appropriately, especially in glioma patients. Marcuse et al. found that 2% of brain tumor patients had NCSE (24 out of 1,101 patients); however, the study included all types of brain tumors [16].

Therefore, the current study aimed to investigate and demonstrate the epidemiology and features of NCSE in the glioma patient population. We hypothesized that NCSE may be underestimated because symptoms of NCSE are often difficult to recognize, especially in glioma patients. To achieve this objective, we retrospectively investigated consecutive glioma patients at our institution and explored NCSE complications in these patients. Additionally, using the Karnofsky Performance Status Scale (KPS), a well-established and widely used neurological impairment assessment tool that reflects the Activity of Daily Living (ADL) [17], we evaluated the impact of NCSE on the lives of glioma patients. The findings of this study may aid in the prompt treatment initiation for NCSE in glioma patients through accurate disease diagnosis, thereby helping clinicians maximize ADL for glioma patients by reducing the distress caused by NCSE complications.

## Methods

We retrospectively reviewed the cases of 108 consecutive patients (63 males and 45 females) with supratentorial glioma who underwent their first operation between April 2013 and May 2019 at Tokyo Medical and Dental University. There were no exclusion criteria. The diagnosis of glioma was confirmed pathologically according to the 2007 World Health Organization (WHO) Classification of Tumors of the Central Nervous System between 2013 and 2016 and the 2016 WHO Classification of Tumors of the Central Nervous System between 2016 and 2019 [18, 19]. We defined low grade glioma as WHO grade I and grade II glioma. We investigated the hospital records of 108 glioma patients, including age, biological sex, tumor location, genetic information of glioma (status of isocitrate dehydrogenase 1 mutation, IDH1-R132H mutation), treatment for glioma, and perioperative anti-seizure medication (ASM) use. The sample size was set to exceed 88 participants in total to yield statistical power of 0.80 with effect size 0.3 for between group analysis (G*power 3.1.9.7).

We selected patients who had been diagnosed with TRE and NCSE from 108 glioma patients. To establish the relationship between glioma patient background and TRE, Fisher’s exact tests were performed, and odds ratios were calculated. The Kruskal–Wallis test was used for glioma location analysis. For NCSE patients, we extracted more detailed information, including (1) seizure type at NCSE onset, (2) temporal course of NCSE (glioma surgery to NCSE symptom onset, NCSE symptom onset to NCSE diagnosis confirmation by electroencephalogram (EEG), treatment period, and NCSE duration), (3) ASM treatment, and (4) KPS before and after NCSE. To validate KPS changes induced by NCSE episodes, we analyzed these changes using paired Student’s t-test. Paired Student t-test was selected as the collected data was assumed to follow the normal distribution (Shapiro–Wilk test, p = 0.50, performed with EZR, a modified version of R commander) [20]. The mean follow-up period and age at surgery of glioma patients with and without NCSE were compared using the Mann–Whitney U test. The Kruskal–Wallis test was also conducted to analyze the difference of glioma location between these two groups. Statistical analysis was determined that they were significant when the p-value was < 0.05. All statistical analyses were performed using GraphPad Prism 8.4.3.

NCSE was diagnosed based on sudden consciousness deficiency episodes, EEG, and the exclusion of other differential diagnoses. EEG results were judged by two or more epileptologists based on periodic discharge, improvement by ASM injection on EEG, and changes over time observed by continuous video-monitored EEG and/or repeated EEG. In addition, computed tomography (CT), magnetic resonance imaging (MRI), and blood tests were performed to exclude tumor progression, stroke, syncope, metabolic syndrome, infection, and other plausible causes that can induce instant consciousness impairment. Furthermore, we confirmed diagnosis based on the efficiency of ASMs in the recovery of consciousness.

We treated NCSE following the treatment guideline for SE that is recommended in the Clinical Practice Guidelines for Epilepsy 2010 and in 2018 published by the Japan Epilepsy Society [21, 22]. These guidelines recommend the staged approach treatment, which includes stage one (benzodiazepine intravenous injection), stage two (phenytoin, phenobarbital, midazolam, or levetiracetam injection), and stage three (general anesthesia using propofol, midazolam, thiopental, or thiamylal). We decided whether the treatment was a success or failure based on EEG improvement because NCSE does not present with obvious motor symptoms.

To confirm NCSE diagnosis, two or more epileptologists retrospectively reviewed the EEG of NCSE patients following the modified Salzburg consensus criteria (mSCC). These criteria have been reported as useful in the diagnosis of NCSE [23].

This study was approved by the Ethics Committee of the Tokyo Medical and Dental University (M2000-1307, 9/25/2012). Written informed consent was obtained from all patients and/or their guardians.

## Results

### Patient characteristics

The characteristics of the 108 glioma patients are shown in Table 1. Patients underwent tumor resection surgery or tumor biopsy at a median age of 62.5 years. Two patients (1.9%) had WHO grade I glioma, 17 patients (16%) had WHO grade II glioma, 26 patients (24%) had WHO grade III glioma, and 63 patients (58%) had WHO grade IV glioma. The pathology for WHO grade I glioma was pilocytic astrocytoma (2 patients). WHO grade II glioma were diffuse astrocytoma (5 patients), oligodendroglioma (11 patients), and low-grade glioma (1 patient). WHO grade III glioma were anaplastic astrocytoma (17 patients), anaplastic oligodendroglioma (8 patients), and anaplastic pleomorphic xanthoastrocytoma (1 patient). WHO grade IV glioma included glioblastoma (60 patients), diffuse midline glioma (2 patients), and gliosarcoma (1 patient). IDH1-R132H mutation was examined in all cases, and 31 patients (29%) had this mutation. Ninety-three patients (86%) underwent radiotherapy, and 85 patients (79%) were treated with chemotherapy. Eighty-four patients (78%) had started receiving an ASM perioperatively for prevention of epileptic seizures.

**Table 1:** Glioma patient characteristics

The number of patients	Total	TRE	NCSE	
	108	61	5	
		56%	4.6%	
Age at glioma surgery (median (range), years old)	62.5 (5–91)	53 (5–91)	45 (41–83)	
follow up period (median, months)	14.6	17	12.7	
Biological sex				
Male	63 (58%)	39 (64%)	3 (60%)	
Female	45 (42%)	22 (36%)	2 (40%)	
WHO grade				
I	2 (1.9%)			
II	17 (16%)	14 (23%)	1 (20%)	
III	26 (24%)	17 (28%)	2 (40%)	
IV	63 (58%)	30 (49%)	2 (40%)	
IDH1-R132H mutation				
Present	31 (29%)	21 (34%)	2 (40%)	
Absent	77 (71%)	40 (66%)	3 (60%)	
Glioma’s main location				
Temporal lobe	27 (25%)	15 (25%)	0 (0%)	
Frontal lobe	44 (41%)	28 (46%)	1 (20%)	
Parietal lobe	21 (19%)	13 (21%)	3 (60%)	
Others	16 (15%)	5 (8.2%)	1 (20%)	
Treatment for glioma				
Radiotherapy	93 (86%)	50 (82%)	4 (80%)	
Chemotherapy	85 (79%)	43 (70%)	3 (60%)	
Perioperative ASM use				
Yes	84 (78%)	49 (80%)	4 (80%)	
No	24 (22%)	12 (20%)	1 (20%)	

*TRE* Tumor-related epilepsy, *NCSE* nonconvulsive status epilepticus, *IDH1-R132H* isocitrate dehydrogenase R132H, *ASM* anti-seizure medication

### Patients with TRE and NCSE

Among 108 glioma patients, 61 patients (56%) showed TRE during the follow-up period. TRE occurrence was significantly associated with chemotherapy only (odds ratio 0.2844, *p* = 0.019, Supplementary Table 1).

Five out of 108 glioma patients (4.6%) were diagnosed with NCSE (three males and two females; Table 1). The median age at the time of glioma surgery was 45 years (range 41–83 years). One patient had WHO grade I glioma (oligodendroglioma), two patients had WHO grade III glioma (anaplastic astrocytoma and anaplastic pleomorphic xanthoastrocytoma), and two patients had WHO grade IV glioma (glioblastoma). Two patients had the IDH1-R132H mutation. Glioma was located mainly in the parietal lobe in three of five patients. There were no significant relationships between NCSE comorbidity and patient characteristics (Supplementary Table 2). The mean follow up period and the mean age at glioma surgery were not significantly different between glioma patients with NCSE and without NCSE (Mann–Whitney U test, p = 0.95 and 0.90 respectively). Glioma location was not significantly different between the two groups (The Kruskal–Wallis test, p = 0.20).

NCSE started as a sudden deficiency in consciousness without prominent motor seizures in four patients (Table 2). Case 4 had preceding convulsive SE from focal to bilateral tonic–clonic seizure (FBTCS), and convulsive SE subsequently progressed to NCSE. EEG (video-monitored continuous EEG and/or repeated EEG) was performed in all five patients to diagnose NCSE.

**Table 2:** Characteristics of glioma patients with TRE or NCSE

	Case 1	Case 2	Case 3	Case 4	Case 5
Age(years)	44	83	72	45	41
Biological sex	F	M	F	M	M
Histology	AA	GBM	GBM	APX	O
WHO grade	III	IV	IV	III	II
IDH1-R132H mutation	Present	Absent	Absent	Absent	Present
Location	Right frontal	Left parieto-frontal	Left parietal	Third ventricle	Left parieto-occipital
Initial TRE seizure type	NCSE	NCSE	FMS	FBTCS	NCSE
Period between glioma surgery and NCSE symptom onset (days)	2	Before glioma surgery	210	871	56
Period between NCSE symptom onset and NCSE diagnosis confirmation by EEG (days)	0	0	0	11	2
Treatment for NCSE	Stage 2	Stage 2	Stage 2	Stage 2	Stage 2
Treatment initiation after NCSE symptom onset (days)	0	0	0	0	2
Needed time for impaired consciousness recovery by NCSE (days)	4	8	11	34	8
Pre NCSE ASM	LEV	None	LEV	LEV	LEV
Post NCSE ASM	LEV(dose up)	VPA	LEV(dose up)	LEV(dose up), PER	LEV PER
Pre NCSE KPS	90	70	70	40	60
Post NCSE KPS	50	30	20	30	40
Follow up period (month)	77.1	8.8	12.7	32.3	3.2

*Pre NCSE ASM* anti-seizure medication which were used before hospitalization due to NCSE, *Post NCSE ASM* anti-seizure medication which were used at the recovery from NCSE, *Pre NCSE KPS* Karnofsky performance status scale before hospitalization due to NCSE, *Post NCSE KPS* Karnofsky performance status scale at discharge from the hospitalization due to NCSE

Biological sex: *F* female, *M* male, Histology: *AA* anaplastic astrocytoma, *GBM* multiform glioblastoma, *APX* anaplastic pleomorphic xantoastrocytoma, *O* oligodendroglioma. *Initial TRE seizure type* Initial tumor related epilepsy seizure type: *NCSE* non-convulsive status epilepticus, *FMS* focal motor onset with awareness, *FBTCS* focal to bilateral tonic–clonic seizure, *EEG* electroencephalogram, *ASM* anti-seizure medication, Pre NCSE ASM anti-seizure medication which were used before NCSE episode: *LEV* levetiracetam, *VPA* valproic acid, *PER* perampanel

Four of five patients with NCSE had NCSE episodes after tumor surgery; however, the time between glioma surgery and NCSE symptom onset varied (Table 2). One patient had NCSE over two years after tumor surgery, while another patient had NCSE two days after tumor surgery.

After the diagnosis of NCSE, ASM treatment was initiated on the same day as NCSE symptom onset in three cases (Table 2). In case 4, the progression from convulsive SE required stage three treatment for the initial convulsive SE. Therefore, we found that NCSE progression depended on prolonged consciousness deficiency after extubation and EEG findings. This explains the delay in diagnosis (11 days after extubation) in case 4. Case 5 was a patient who was transferred from another hospital two days after the onset of NCSE symptoms; therefore, diagnosis and treatment initiation were delayed. NCSE was managed by stage two treatments in all five patients.

All patients, except case 2, were administered prophylactic ASMs at the onset of NCSE (Table 2). Case 2 showed NCSE before the initiation of preventative ASMs during the perioperative period. After NCSE episodes, three patients needed an additional ASM, and the other two patients needed an increased dose of the same ASMs.

### Karnofsky performance status scale and NCSE

**Fig. 1:**
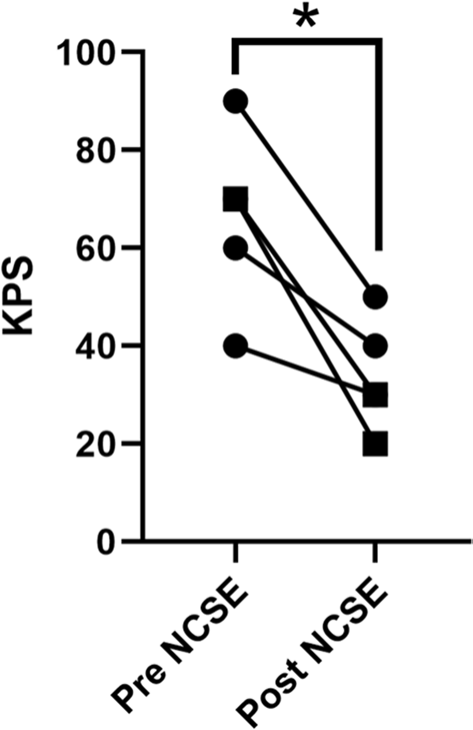
KPS changes before and after NCSE. Pre KPS is prehospital KPS just before an NCSE episode. Post KPS is KPS at discharge from the hospitalization for NCSE. Prehospital mean KPS was 66, mean KPS at discharge was 34. The mean KPS scores were compared between Pre KPS and Post KPS using paired Student t-test. *p = 0.0121. Black squares represent cases of patients aged over 70 years

We investigated the KPS scores of all five NCSE patients before and after NCSE episodes to understand the effect of NCSE on ADL. KPS significantly declined at discharge compared with that before hospitalization due to NCSE (34 versus 66, *p* = 0.0121) (Fig. 1). KPS decline was high (> 50% decline) in two older people (> 70 years, black square in Fig. 1), although significant difference was not found (Mann–Whitney U test, p = 0.30).

### EEG findings and modified Salzburg consensus criteria (mSCC)

To confirm NCSE diagnosis, two or more epileptologists analyzed the EEG of five NCSE-diagnosed patients retrospectively according to mSCC (Table 3). EEG showed rhythmic activity > 0.5 Hz and spatiotemporal evolution in all patients with NCSE. In contrast, only one patient showed typical epileptiform discharge at above 2.5 Hz. Consciousness deficiency and EEG features improved with intravenous ASM in all five patients.

**Table 3:** Five NCSE patients’ EEG and clinical data reviewed by modified Salzburg Consensus Criteria for NCSE

		Case 1	Case 2	Case 3	Case 4	Case 5
EEG data	Epileptiform discharge > 2.5 Hz	n	n	n	n	y
	Spatio-temporal evolution	y	y	y	y	y
	Rhythmic activity > 0.5 Hz	y	y	y	y	y
	Improvement of clinical and EEG features with IV ASM	y	y	y	y	y
Clinical data	Subtle clinical ictal phenomenon	n	n	y	n	n
	Transition from premorbid to current ill state within minutes to hours	y	y	y	n	y
	Patient did not improve significantly in last minutes to hours, apart from waxing and waning	y	y	y	y	y
	No information from brain imaging sufficiently explaining EEG pattern	y	y	y	y	y
	No metabolic/toxicological derangement sufficiently explaining EEG	y	y	y	y	y

*NCSE* non-convulsive status epilepticus, *EEG* electroencephalogram, *IV ASM* intravenous anti-seizure medication

### Case presentation: 41 years old male (Case 5)

**Fig. 2:**
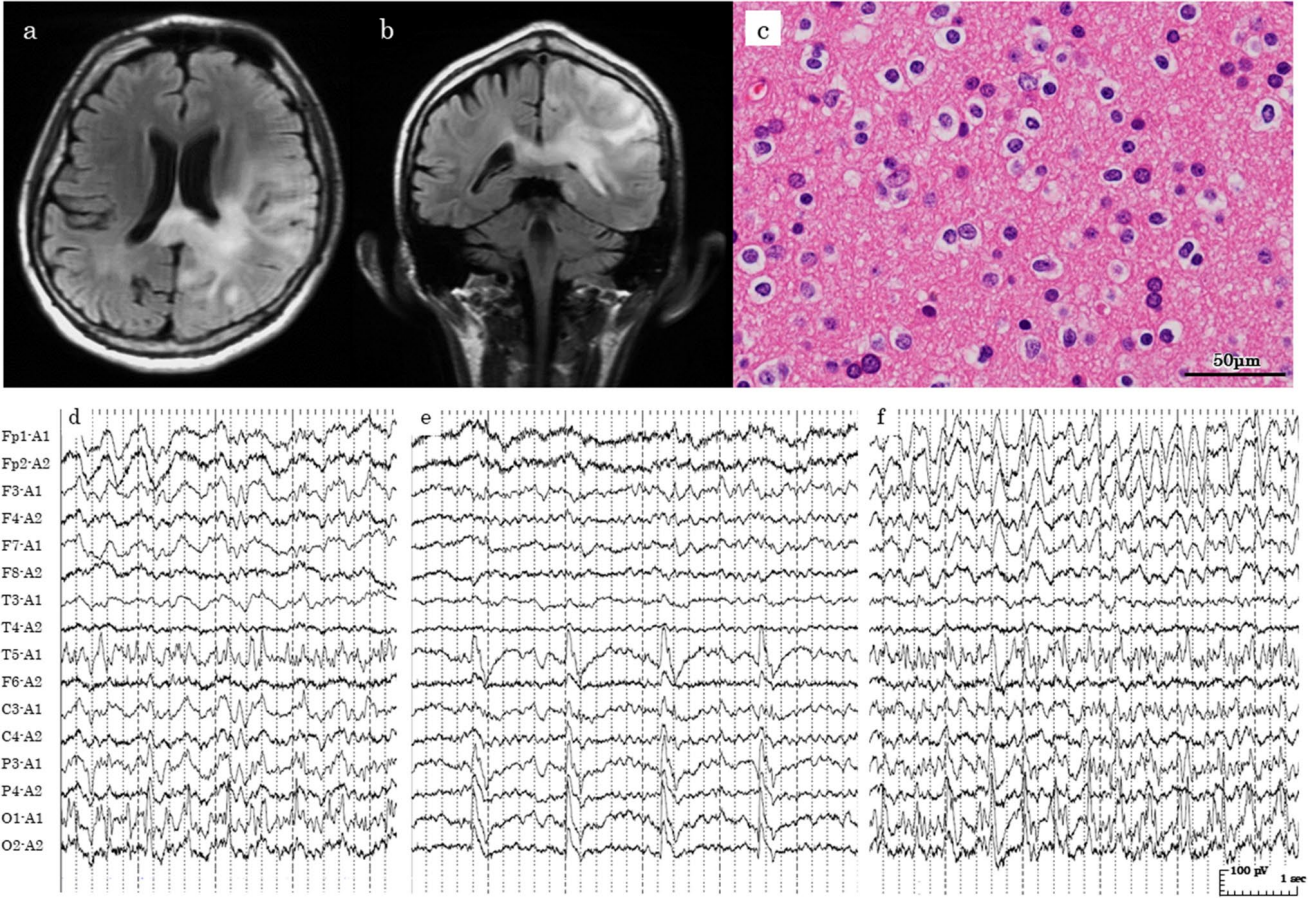
MRI, histology and EEG of Case 5. **a**, **b** Preoperative axial and coronal FLAIR showed a hyperintense lesion on the left parieto-occipital lobe (**a** axial plane, **b** coronal plane). **c** Hematoxylin and eosin staining (HE) of the tumor. Oligodendroglioma was confirmed pathologically. **d** Continuous epileptiform discharge with above 2.5Hz frequency mainly in the left occipital region during more than 10 s (O1, T5). **e** Rhythmic activity with ˃0.5 Hz frequency mainly in the occipito-temporal region (O1, T5). **f** Spatiotemporal evolution of epileptic discharge into the parietal region (C3, P3)

The NCSE episode in case 5 is described below. A 41-year-old man initially presented with hemiparesis. Preoperative brain MRI fluid-attenuated inversion recovery (FLAIR) showed a hyperintense lesion in the left parieto-occipital lobe (Fig. 2a and b). The patient underwent a tumor biopsy. Oligodendroglioma (WHO grade II) was pathologically confirmed by a neuro-oncologist and a pathologist following the 2016 WHO Classification of Tumors of the Central Nervous System (Fig. 2c). The patient had been taking levetiracetam preventatively from the perioperative period. After the biopsy, the patient received localized external beam radiation therapy (1.8 Gray per day, 30 days, 54 Gray in total). There were no epileptic seizures or consciousness deficiencies during the radiation treatment course. Fifty-six days after surgery, the patient suddenly fell down in the station while on the way home and was taken to another hospital. The patient had a consciousness deficiency [Glasgow Coma Scale (GCS) 14 (E4V4M6) and Japan Coma Scale (JCS) I-2]; afterward, the patient was transferred to Tokyo Medical and Dental University Hospital for two days. The patient still had a consciousness deficiency (GCS 15 (E4V5M6), JCS I-1) without motor seizures. Brain imaging (MRI and CT) revealed no intracranial hematoma, cerebral infarction, tumor necrosis, or tumor progression. Systemic examinations, including vital signs, blood tests, electrocardiogram, and chest X-ray, excluded infections, metabolic abnormalities, or other causes that could induce sudden onset and prolonged consciousness deficiency. We conducted EEG which showed (1) continuous epileptiform discharge above 2.5 Hz mainly in the left occipito-temporal region (O1, T5) during > 10 s (Fig. 2d), (2) rhythmic activity above 0.5 Hz mainly in the left occipital region (O1, T5) (Fig. 2e), and (3) spatiotemporal evolution of epileptic discharge into the left parietal region (C3, P3) (Fig. 2f). Therefore, we diagnosed NCSE on the same day of hospitalization at our hospital and started diazepam (stage one treatment), and phenytoin, levetiracetam, and perampanel (stage two treatment). He recovered from consciousness deficiency five days after the start of treatment. Furthermore, EEG findings improved after two weeks. He was discharged with a drop in the KPS score from 60 to 40.

## Discussion

This is the first study to reveal the comorbidity rate and features of NCSE in glioma patients. Our study revealed three important findings. First, 4.6% of consecutive glioma patients had NCSE. This shows that NCSE is not a rare complication in glioma patients; instead, the frequency is more than twice of that of general brain tumor patients, according to a previous study [16]. Second, ADL significantly decreased after NCSE episodes. Third, mSCC could help in NCSE diagnosis in glioma patients, especially by following EEG instructions. These findings emphasize the importance of managing NCSE in glioma patients. At the same time, initiative-taking EEG, especially when analyzed by mSCC, may facilitate accurate NCSE diagnosis and prompt initiation of treatment, which can lead to improved ADL in glioma patients.

In particular, the high prevalence of NCSE as a complication of glioma should receive increased attention. NCSE has been associated with brain tumors and 4–12% of NCSE patients have been reported to have a pre-existing brain tumor [15]. In addition, Giovannini et al. reviewed SE population and reported that 19 (4.3%) patients had glioma related NCSE [24]. The frequency revealed by this present study matches these data; however, previous studies investigated the SE population and not the glioma population. Our focus was on NCSE as one of the comorbidities of glioma; therefore, 4.6% of patients is an intuitively useful number when you diagnose NCSE in glioma patient population. This difference in population focus characterizes the novelty of our study.

Another important finding of this study is that NCSE had a significant negative effect on ADL. Previous clinical studies showed that NCSE neurological deficits occur in 10 to 40% of patients [25], with a mortality rate of 18 to 48% [16, 26–28]. Horvath et al. reported that NCSE was associated with mortality during long-term follow-up (odds ratio 9.86) [29]. Additionally, Dominguez et al. reported that worse outcomes were observed in patients with NCSE who had been overlooked and had not been treated properly [30]. In addition to these clinical studies, one preclinical study supports the poor outcomes of NCSE by demonstrating histological neural damage and glial reactive changes caused by NCSE using rodent models [31]. The significant KPS disturbance induced by NCSE in this study revealed the critical negative effects of NCSE on ADL in patients with glioma. Interestingly, two elderly patients [Case 2 (83 years old) and Case 3 (72 years old)] showed a relatively large decrease in KPS (Mann–Whitney U test, p = 0.30), although there was no significant difference between those over 70 and under 70 years old. The prognosis of NCSE was reported to be associated with age and the elderly people are known as one of the groups who shows higher mortality after SE [32]. However, to our knowledge, there is no revealing the relationship between age and the prognosis of NCSE in glioma population. Further accumulation of cases is needed to clarify the effect of NCSE on ADLs in the elderly glioma patients.

The most problematic dilemma is the difficulty in prompt and accurate diagnosis of NCSE due to the absence of established diagnostic criteria. However, promising diagnostic criteria for NCSE have recently gained attention. The Salzburg consensus criteria were reported in 2013 with 97.7% sensitivity, 89.5% specificity, and a 92.5% overall accuracy rate [33]. In 2015, mSCC was developed upon the improving Salzburg consensus criteria; the criteria proposed a reduction of false positives (from 28 to 0%) [23]. Regarding practicality in clinical situations, Krogstad et al. showed that amateurs diagnosed NCSE with EEG as accurately as experts when they followed mSCC [34]. In terms of clinical outcomes, prompt diagnosis of NCSE according to mSCC leads to long-term survival after NCSE [35]. In this study, we retrospectively confirmed that all NCSE patients could be diagnosed by the mSCC. EEG results can be changed by glioma and glioma surgery. However, mSCC can detect NCSE distinctly even in glioma patients who already have abnormal EEG because some of the EEG feature definitions in mSCC focus on longitudinal EEG changes. To initiate prompt treatments for NCSE according to the mSCC and observed seizure symptoms, EEG, especially continuous video-monitored EEG that can capture EEG changes (including ASM response) over time, should be performed promptly when NCSE is suspected as a differential diagnosis for sudden consciousness impairment in glioma patients.

However, treatments for NCSE remain under discussion. There are no clear answers to how NCSE should be treated because there are no established treatment guidelines for NCSE. There are a few studies of treatment outcomes in NCSE. Seizures were reported to be controlled in 92% of NCSE patients and clinical symptoms were improved by existing ASM treatments as a complication of brain tumor [16]. The Treatment of Recurrent Electrographic Nonconvulsive Seizures Trial (TRENdS trial), a prospective, randomized, blinded, and controlled trial for evaluating ASMs for nonconvulsive seizures, demonstrated non-inferiority of lacosamide to fosphenytoin (*p* = 0.02) and showed that NCSE was controlled in 63.3% patients who were refractory to the two ASMs [36]. Bravo et al. reviewed the response rate to ASM in NCSE patients and reported response rates between 17 and 100%, depending on the ASMs or studies [37]. In this present study, the treatment for SE recommended in the Clinical Practice Guidelines for Epilepsy published by the Japan Epilepsy Society was also effective in the management of NCSE, and stage two treatment (ASM injection) was able to control NCSE in all five patients. However, treatment initiation on the same day of NCSE onset started did not sufficiently prevent ADL disruption. Therefore, we need to accumulate NCSE treatment experience to establish systemic NCSE treatment guidelines for better outcomes.

This study had two limitations including the small sample size and retrospective study design. We attempted to describe the significantly related background of NCSE; however, it was difficult to show statistical significance because of the small sample size. In addition, a future prospective study is essential to demonstrate the risk factors for NCSE.

## Conclusion

Glioma patients showed a higher prevalence of NCSE than the general brain tumor population. In addition, there was a significant KPS decline after NCSE episodes. Prompt EEG examinations and analysis following mSCC may lead to an accurate diagnosis of NCSE. As a result, immediate treatment of NCSE can be initiated to maximize the ADL of glioma patients.

## Supplementary Information

Supplementary file1 (XLSX 9 KB)

Supplementary file2 (XLSX 10 KB)

## Data Availability

The datasets generated and/or analyzed during the study are available from the corresponding author upon reasonable request.

## References

[1] Ostrom QT, Bauchet L, Davis FG, Deltour I, Fisher JL, Langer CE, Pekmezci M, Schwartzbaum JA, Turner MC, Walsh KM, Wrensch MR, Barnholtz-Sloan JS (2016). The epidemiology of glioma in adults: a “state of the science” review. Neuro Oncol.

[2] Wen PY, Weller M, Lee EQ, Alexander BM, Barnholtz-Sloan JS, Barthel FP, Batchelor TT, Bindra RS, Chang SM, Chiocca EA, Cloughesy TF, DeGroot JF, Galanis E, Gilbert MR, Hegi ME, Horbinski C, Huang RY, Lassman AB, Le Rhun E, Lim M, Mehta MP, Mellinghoff IK, Minniti G, Nathanson D, Platten M, Preusser M, Roth P, Sanson M, Schiff D, Short SC, Taphoorn MJB, Tonn JC, Tsang J, Verhaak RGW, von Deimling A, Wick W, Zadeh G, Reardon DA, Aldape KD, van den Bent MJ (2020). Glioblastoma in adults: a Society for Neuro-Oncology (SNO) and European Society of Neuro-Oncology (EANO) consensus review on current management and future directions. Neuro Oncol.

[3] van Breemen MS, Wilms EB, Vecht CJ (2007). Epilepsy in patients with brain tumours: epidemiology, mechanisms, and management. Lancet Neurol.

[4] Thijs RD, Surges R, O’rien TJ, Sander JW (2019). Epilepsy in adults. Lancet.

[5] Nowell M, Miserocchi A, McEvoy AW (2015). Tumors in epilepsy. Semin Neurol.

[6] Barragán-Ardila J, Mayor LC, Mancera J, Martínez Micolta P (2022). Ictal paraphasia as an atypical manifestation of temporal lobe epilepsy. Neurocase.

[7] Chen DY, Chen CC, Crawford JR, Wang SG (2018). Tumor-related epilepsy: epidemiology, pathogenesis and management. J Neurooncol.

[8] Marcus LP, McCutcheon BA, Noorbakhsh A, Parina RP, Gonda DD, Chen C, Chang DC, Carter BS (2014). Incidence and predictors of 30-day readmission for patients discharged home after craniotomy for malignant supratentorial tumors in California (1995–2010). J Neurosurg.

[9] Atmaca MM, Bebek N, Baykan B, Gökyiğit A, Gürses C (2017). Predictors of outcomes and refractoriness in status epilepticus: a prospective study. Epilepsy Behav.

[10] Shorvon S, Sen A (2020). What is status epilepticus and what do we know about its epidemiology?. Seizure.

[11] Rosenow F, Hamer HM, Knake S (2007). The epidemiology of convulsive and nonconvulsive status epilepticus. Epilepsia.

[12] Knake S, Rosenow F, Vescovi M, Oertel WH, Mueller HH, Wirbatz A, Katsarou N, Hamer HM, Status Epilepticus Study Group Hessen (SESGH) (2001). Incidence of status epilepticus in adults in Germany: a prospective, population-based study. Epilepsia.

[13] Dupont S, Kinugawa K (2020). Nonconvulsive status epilepticus in the elderly. Rev Neurol.

[14] Brauchitsch SV, Strzelczyk A, Rosenow F, Neuhaus E, Dubinski D, Steinbach JP, Voss M (2022). High end-of-life incidence of seizures and status epilepticus in patients with primary and secondary brain tumors. J Neurooncol.

[15] Casazza M, Gilioli I (2011). Non-convulsive status epilepticus in brain tumors. Neurol Sci.

[16] Marcuse LV, Lancman G, Demopoulos A, Fields M (2014). Nonconvulsive status epilepticus in patients with brain tumors. Seizure.

[17] Milstein JM, Cohen ME, Sinks LF (1985). The influence and reliability of neurologic assessment and Karnofsky performance score on prognosis. Cancer.

[18] Louis DN, Ohgaki H, Wiestler OD, Cavenee WK, Burger PC, Jouvet A, Scheithauer BW, Kleihues P (2007). The 2007 WHO classification of tumours of the central nervous system. Acta Neuropathol.

[19] Louis DN, Ohgaki H, Perry A, Reifenberger G, Deimling AV, Figarella-Branger D, Cavenee WK, Ohgaki H, Wiestler OD, Kleihues P, Ellison DW (2016). The 2016 World Health Organization classification of tumors of the central nervous system: a summary. Acta Neuropathol.

[20] Kanda Y (2013). Investigation of the freely available easy-to-use software ‘EZR’ for medical statistics. Bone Marrow Transplant.

[21] Japanese Society for Neurology (2010). Clinical practice guidelines for epilepsy 2010.

[22] Japanese Society for Neurology (2018). Clinical practice guidelines for epilepsy 2018.

[23] Leitinger M, Beniczky S, Rohracher A, Gardella E, Kalss G, Qerama E, Höfler J, Lindberg-Larsen AH, Kuchukhidze G, Dobesberger J, Langthaler PB, Trinka E (2015). Salzburg consensus criteria for non-convulsive status epilepticus—Approach to clinical application. Epilepsy Behav.

[24] Giovannini G, Pasini F, Orlandi N, Mirandola L, Meletti S (2019). Tumor-associated status epilepticus in patients with glioma: Clinical characteristics and outcomes. Epilepsy Behav.

[25] Trinka E, Cock H, Hesdorffer D, Rossetti AO, Scheffer IE, Shinnar S, Shorvon S, Lowenstein DH (2015). A definition and classification of status epilepticus—Report of the ILAE Task Force on Classification of Status Epilepticus. Epilepsia.

[26] Shneker BF, Fountain NB (2003). Assessment of acute morbidity and mortality in nonconvulsive status epilepticus. Neurology.

[27] Baysal-Kirac L, Cakar MM, Altiokka-Uzun G, Guncan Z, Guldiken B (2021). Electroclinical patterns in patients with nonconvulsive status epilepticus: etiology, treatment, and outcome. Epilepsy Behav.

[28] Yuan F, Yang F, Li W, Yang X, Gao Q, Bi L, Jiang Y, Jiang W (2018). Nonconvulsive status epilepticus after convulsive status epilepticus: clinical features, outcomes, and prognostic factors. Epilepsy Res.

[29] Horváth L, Fekete I, Molnár M, Válóczy R, Márton S, Fekete K (2019). The outcome of status epilepticus and long-term follow-up. Front Neurol.

[30] Domínguez AG, Mateo Montero RC, Díaz Cid A, Mazarro AJP, Bailly-Bailliere IR, Landete IMS, Palomeque GM (2021). Salzburg criteria, a useful tool in non-convulsive status epilepticus diagnosis: a retrospective study. Clin EEG Neurosci.

[31] Avdic U, Ahl M, Chugh D, Ali I, Chary K, Sierra A, Ekdahl CT (2018). Nonconvulsive status epilepticus in rats leads to brain pathology. Epilepsia.

[32] Aslan-Kara K, Demir T, Satılmış Ü, Peköz T, Bıçakcı Ş, Bozdemir H (2022). Prognostic indicators of non-convulsive status epilepticus in intensive care unit. Acta Neurol Belg.

[33] Leitinger M, Trinka E, Gardella E, Rohracher A, Kalss G, Qerama E, Höfler J, Hess A, Zimmermann G, Kuchukhidze G, Dobesberger J, Langthaler PB, Beniczky S (2016). Diagnostic accuracy of the Salzburg EEG criteria for non-convulsive status epilepticus: a retrospective study. Lancet Neurol.

[34] Krogstad MH, Høgenhaven H, Beier CP, Krøigård T (2019). Nonconvulsive status epilepticus: validating the Salzburg criteria against an expert EEG examiner. J Clin Neurophysiol.

[35] Monsson OS, Roberg LE, Gesche J, Beier CP, Krøigård T (2022). Salzburg consensus criteria are associated with long-term outcome after non-convulsive status epilepticus. Seizure.

[36] Husain AM, Lee JW, Kolls BJ, Hirsch LJ, Halford JJ, Gupta PK, Minazad Y, Jones JM, LaRoche SM, Herman ST, Swisher CB, Sinha SR, Palade A, Dombrowski KE, Gallentine WB, Hahn CD, Gerard EE, Bhapkar M, Lokhnygina Y, Westover MB, Critical Care EEG Monitoring Research Consortium (2018). Randomized trial of lacosamide versus fosphenytoin for nonconvulsive seizures. Ann Neurol.

[37] Bravo P, Vaddiparti A, Hirsch LJ (2021). Pharmacotherapy for nonconvulsive seizures and nonconvulsive status epilepticus. Drugs.

